# Spaceborne NO_2_ observations are sensitive to coal mining and processing in the largest coal basin of Russia

**DOI:** 10.1038/s41598-022-16850-8

**Published:** 2022-07-22

**Authors:** Lev D. Labzovskii, Dmitry A. Belikov, Alessandro Damiani

**Affiliations:** 1 R&D Satellite and Observations Group, Netherlands Meteorological Institute (KNMI), De Bilt, The Netherlands; 2 Center for Environmental Remote Sensing, Chiba University, Chiba, 263-8522 Japan

**Keywords:** Environmental sciences, Environmental impact, Atmospheric science

## Abstract

Coal use exacerbates several major environmental problems including build-up of greenhouse gases and air quality deterioration. Although Kuzbass (Siberia) is one of the largest exploited coal basins worldwide, the role of regional coal mining and processing in atmospheric pollution is unknown. We outlined the Kuzbass coal basin by spaceborne night-lights and revealed a regional, long-term tropospheric NO_2_ anomaly (2005–2018) by spaceborne NO_2_ column observations (hereafter ‒ NO_2_). The spatial agreement between NO_2_ and night-lights indicates that the anomaly is attributable to an agglomeration of coal quarries and the cities in Kuzbass, that are heavily reliant on coal. A positive relationship between NO_2_ and interannual coal production suggested that the anomaly was related to coal in Kuzbass; ~ 1.0% of annual coal production increase induced ~ 0.5–0.6% of NO_2_ enhancement. As coal production accelerated since 2010, NO_2_ exhibited strikingly similar annual increases over Kuzbass in 2010–2014 (7%) and 2015–2019 (15%), compared to 2005–2009. Conversely, Siberian cities lacking a coal industry followed the global trend of reducing NO_2_ for the same periods (−5% and −14%, respectively), driven by fuel combustion improvements. Overall, we demonstrated that coal mining, processing and utilization can induce distinct tropospheric NO_2_ anomalies, detectable from space.

## Introduction

The global reliance on coal has triggered infamously adverse environmental effects as coal has become the main contributor of the atmospheric greenhouse gas accumulation and the most atmosphere-polluting source of energy as well^1^. These adverse effects stem from direct emissions of coal mining (scraping and fracturing of coal from ground) as well as from indirect emissions from coal mining (fuel combustion of coal mining machinery), coal processing (production, conversion to coke, use in metal production) and coal transportation (emissions from coal-carrying vehicles)^2^. There is a growing corpus of studies, relying on spaceborne observations to monitor and constrain atmospheric emissions, originating from coal power plants^3–5^. However, only few investigations have considered spaceborne observations of atmospheric byproducts from areas of coal mining and processing. Previous studies have focused on developed countries such as the U.S.^6^ and Australia^7^, but 84% of the world’s coal mining and processing are in emerging and developing economies^8^, following the development classification of the International Monetary Fund^9^. Knowledge of the atmospheric byproducts of coal mining and processing is incomplete for these countries in comparison with developed countries. This is peculiarly undesirable, because officially reported emissions in these countries can be strikingly inaccurate^10–12^.

A growing research interest in Chinese coal point-sources^13^ has provided previously unknown information about coal byproducts in the atmosphere. However, various other emerging or developing countries remain unexplored in this respect. The post-Soviet states (accounting for 7% of global coal production) are the most salient examples of such blind-spots, considering the presence of large coal-producing regions such as Donbass in Ukraine and, especially, Kuzbass in Russia. Kuzbass, located in southwest Siberia, is the largest coal basin in Russia and one of the largest in the world (~ 300 billion tons of accessible reserves), containing 33% of the world’s known coal deposits^14^. Despite the global importance of Kuzbass, only Oparin et al.^15^ have addressed the air pollution over the region by using spaceborne remote sensing of snow cover. Besides their indirect evidence of air pollution, detectable from snow cover in Kuzbass, no other empirical work has attempted to estimate the atmospheric pollution or composition over Kuzbass. The literature contains only fragmented information about the effects of coal mining on the atmosphere over this coal-rich area such as the indication about elevated tropospheric NO_2_ over southwestern Siberia in 2005‒2018, visible from a NO_2_ concentration map of Asia in Jamali et al.^16^ The authors have never addressed this local increase, but such hint about a potential NO_2_ atmospheric enhancement over a major coal basin is intriguing because NO_2_ is not a common indicator of a direct coal mining outgassing. Rather, it is attributed to indirect emissions of coal mining, stemming from fuel combustion, mainly by motor vehicles^17^ but also by the heavy machinery and transportation vehicles involved in the mining, processing, and transportation of coal^18^. In this context, a regional study reported that in Kuzbass, huge amounts of coal are mined (by excavators) and transported (by haul trucks) using heavy machinery that relies on the inefficient combustion of diesel fuel^19^. Given the scales of coal mining in Kuzbass, such machinery generates massive amounts of NO_x_ emissions that might increase tropospheric NO_2_ to levels that are seemingly harmful to respiratory and cardiovascular systems^20^.

For this reason, our study elucidates a potential link between coal mining/processing activities, and atmospheric NO_2_ over Kuzbass by using a set of spaceborne remote sensing observations: NO_2_ tropospheric columns from the Ozone Monitoring Instrument (OMI), night lights from the Operational Line-scan System (OLS), and urban pixels from the Moderate-resolution Imaging Spectroradiometer (MODIS). To this end, we (a) investigated the statistical agreement between NO_2_ and the cluster of anthropogenic activities in Kuzbass (the concentrations of urban areas, industrial clusters, and opencast coal mines in the region); (b) examined the sensitivity of NO_2_ to coal production (from reported data) in Kuzbass; and (c) separately compared the long-term trends of NO_2_ for Kuzbass cities and other cities in Siberia that have no links to the coal industry.

## Results

**Figure 1:**
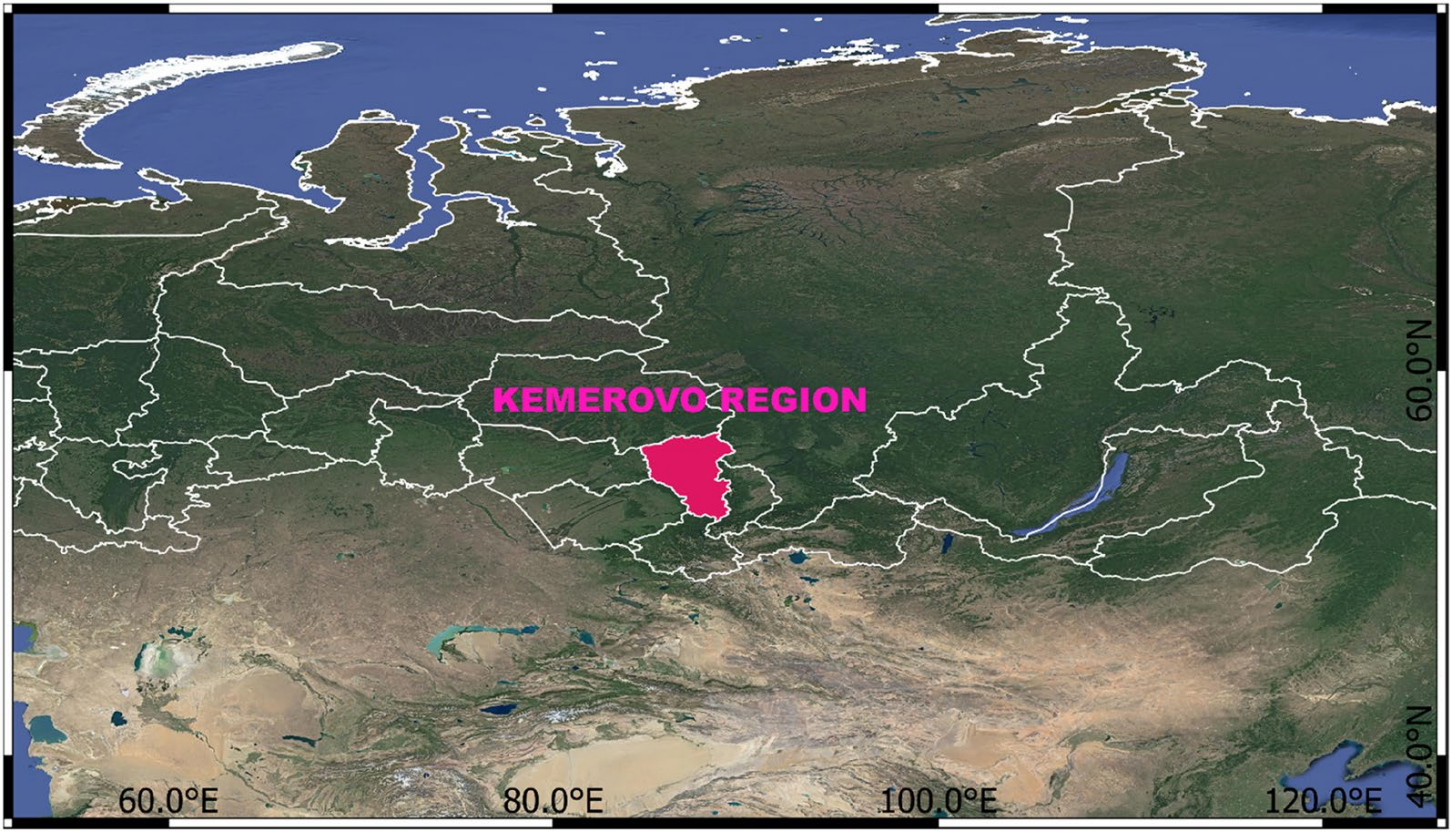
Location of the Kemerovo region (pink area). Top-level administrative boundaries in Russia are shown in white. The satellite map provided by Google Maps is embedded using the QucikMapServices plugin of QGIS software (0.19.29 https://nextgis.com/blog/quickmapservices/).

Kuzbass is located in southwestern Siberia and lies within the Kemerovo administrative region of Russia (Fig. 1), which is specialized in mining and processing industries, occupying ~ 50% of regional economy. Kuzbass covers the area of ~ 26,000 km^14^, contains ~ 300 billion tons of coal^14^, and is responsible for 58% of coal produced in Russia and 70% of exported coal^21^. Most of the coal (63.8%) is mined in open pits^22^, and there are 90 such mines^23^. Compared with other coal-rich regions in Russia, Kuzbass is a hotspot of coal mining, processing and utilization because of its mild climate and near-surface coal seams, which are conductive for open-pit mining and are cost-effective^21^. The adverse environmental impacts of coal-related industry in Kuzbass are clear, including 2.5 billion tons of waste (50% of the solid waste in Russia), with 98% of this waste originated from mining activities^22^.

**Figure 2:**
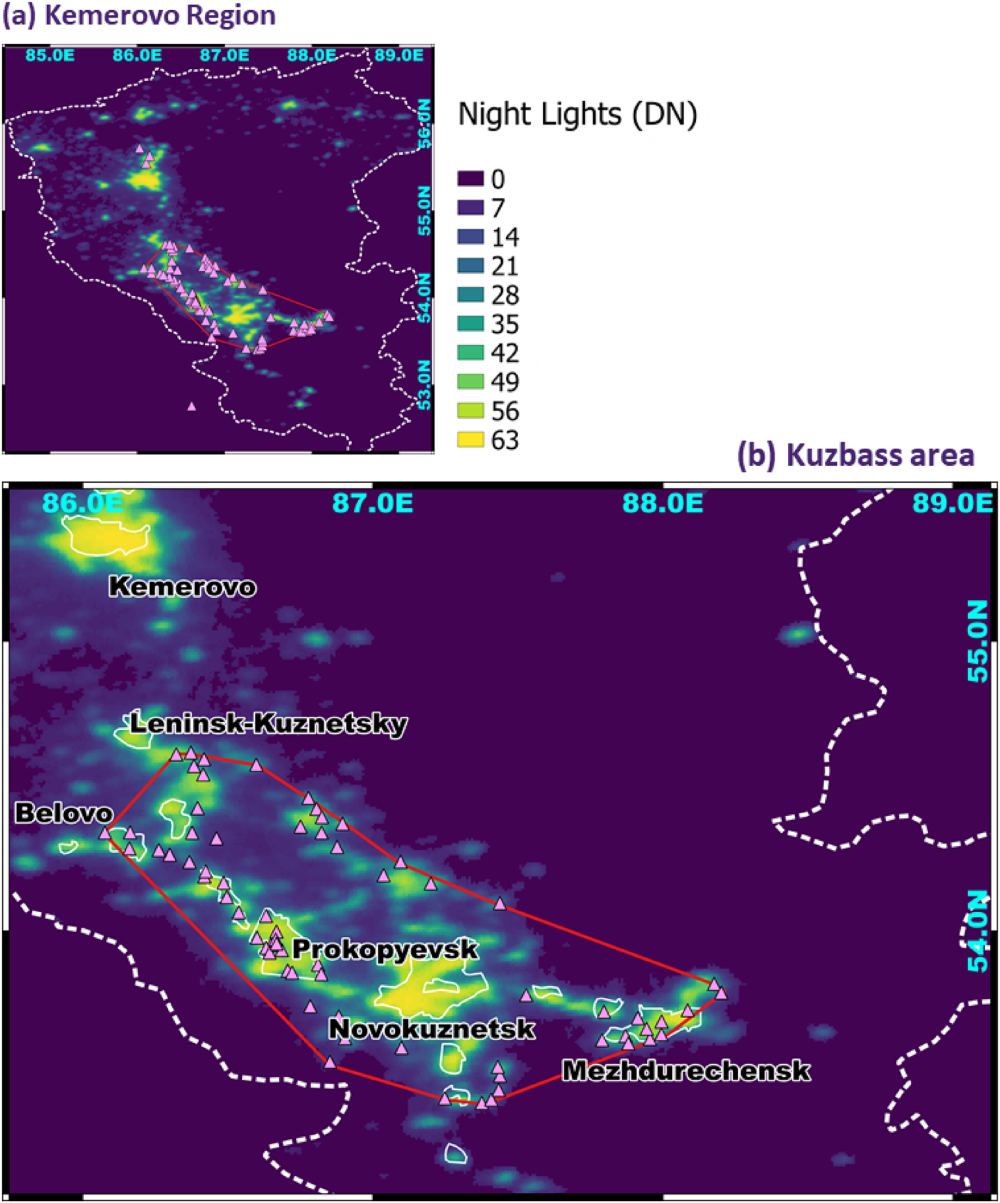
The delineated cluster of coal quarries and large cities, representing Kuzbass, shown on (**a**) the Kemerovo administrative region map (dashed white line) with Kuzbass coal basin (red polygon) and on (**b**) the zoomed-in map of the Kuzbass coal basin (red polygon) with the detected open coal quarries (triangles), MODIS urban pixels (white solid line) and night-lights (black-yellow gradient).

While the boundaries of Kemerovo region are administratively defined (Fig. 1), the boundaries of the Kuzbass basin can vary depending on the application. There is no robust, validated map of Kuzbass coal quarries, compiled by established surveying techniques. Given the small size of the study area and the visual prominence of quarries against the green vegetation of the surrounding taiga ecosystem, we used Google Earth imagery and manually marked the centers of the largest mines in the region (Fig. S2, Supplementary Material). As a result, 81 open coal quarries were outlined (out of 90 reported open quarries^23^). These quarries were used to create a polygon by connecting all the marginal coal quarries within the geographical cluster in the center of Kemerovo region with most identified quarries (68/81) within (red line, Fig. 2). This cluster reflects the heartland of the regional coal basin and is hereafter referred to as Kuzbass.

As shown in Fig. 2, we validated the mapping of mines in Kuzbass by using the latest estimates of cloud-free night lights from OLS measurements, which are a proxy for human activity^24^. Areas with opencast mines operated during the night normally exhibit distinct local increases in the digital number (DN) of night lights^25^. We also used the map of urban areas from MODIS, based on the unique phenology of build-up areas^26^ to distinguish cases of increased night lights originating from urban areas (when the lights align with the MODIS urban map) from cases of increased night lights from a coal mine outside a built-up zone (when there is no overlap of lights with the MODIS urban map). A lack of overlap of night-lights with the urban population data has been previously used to identify night light signals that are unrelated to cities^27^. Figure 2 demonstrates that the mapping of the Kuzbass quarries realistically reflected the regional patterns of urbanization and the coal quarry allocation. The combination of coal mine mapping with night lights and the MODIS urban map (Fig. 2) hints at three broad types of area modified by human activity in Kuzbass: (1) urban areas without mines, identified by strong night lights overlapping with the MODIS urban map. An example is Novokuznetsk, a major regional conurbation with an industrial economy, specializing in metal production^28^, but relatively weak underground coal mining. The second (2) is urban areas that include coal mines. The urban areas are identified as above, but there are also coal mines, identified within the outlined areas. Examples are Mezhdurechensk and Prokopyevsk, cities planned and evolved exclusively as coal mining centers. The third area type is (3) coal mining zones, characterized by moderate night light intensity and the presence of mines but with no overlap with MODIS urban pixels. Note the difference between Kemerovo region (one of Russia’s top-level political divisions, shown in Figs. 1 and 2a) and Kemerovo City, the region’s capital city (an urban area; uppermost city in Fig. 2b).

**Figure 3:**
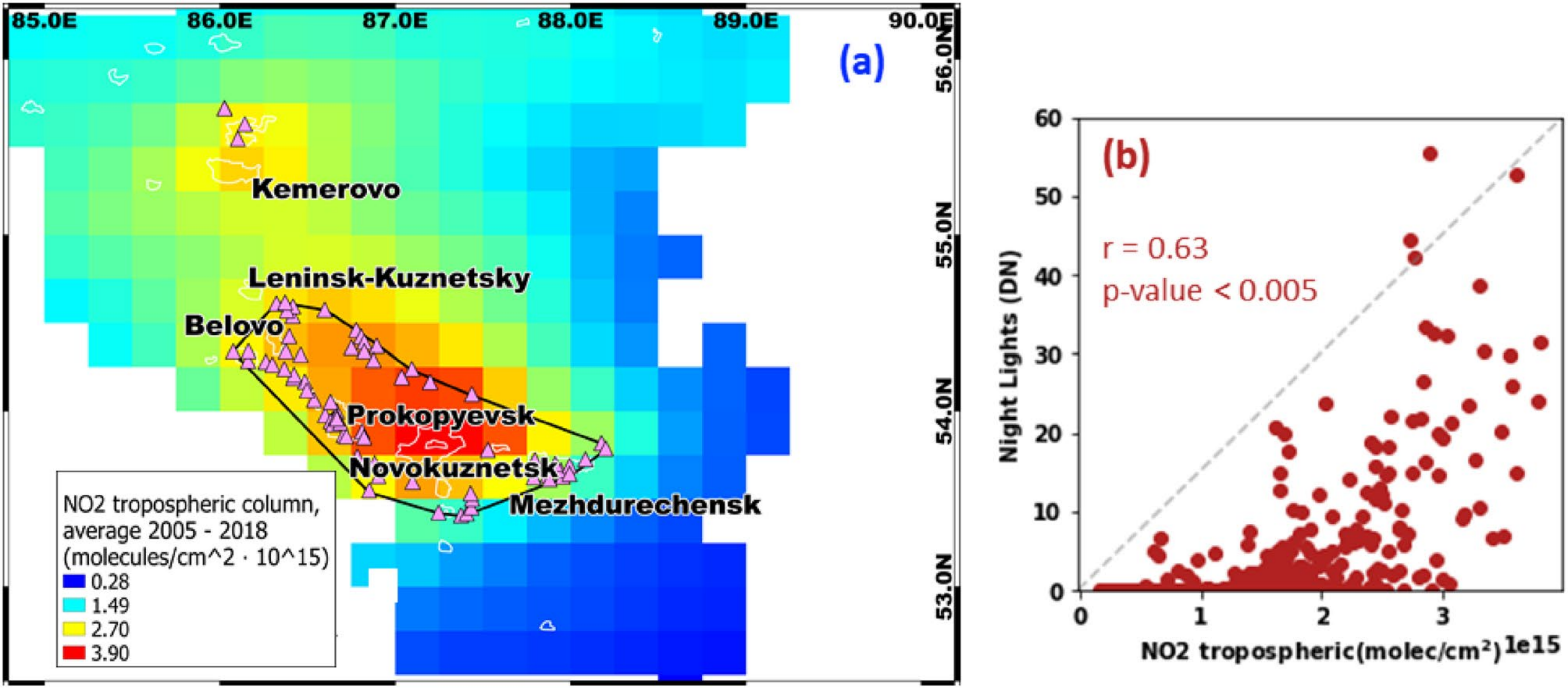
(**a**) Spatial distribution of average NO_2_ tropospheric column (2005–2018) over Kemerovo region and Kuzbass (constrained by black outline), illustrated by the blue-red gradient, coal quarries in the region (triangles) and MODIS urban pixels (white lines); (**b**) Scatter plot of night light (DN) and the corresponding NO_2_ average over Kuzbass (within the polygon, outlined by black in **a**) for each 0.25° × 0.25° pixel. A note: the abrupt change in NO_2_ concentration at the eastern margin of the Kuzbass region (blue color in **a**) is not related to night lights (i.e., anthropogenic activity), but to the local topography. In fact, the eastern margin of the region has the highest elevation in the study area (600‒1050 m above sea level, asl; Fig. S3 in the Supplementary Material). NO_2_ generally accumulates under two topographic conditions: the hindered dispersion of pollutants due to low penetration of winds in a trough, and pollution being trapped near the ground surface by temperature inversions formed by surrounding mountains^44^. The center of Kuzbass (the strongest NO_2_ anomaly, red in **a**) is subjected to these topographic conditions, as it is located in a trough (~ 250 m asl) surrounded by hills (Fig. S3). It is therefore likely susceptible to temperature inversions, but there were no regional meteorological studies to evaluate this hypothesis in detail.

As mentioned, a distinct increase of tropospheric NO_2_ over southwestern Siberia had been evidenced in a previous study^16^, but remained uncommented by its authors. We calculated the average NO_2_ for 2005–2018 and revealed two distinct NO_2_ spatial enhancements (e.g., positive anomalies) over southwestern Siberia as well, namely, over Kemerovo region (Fig. 3a). These two major long-term NO_2_ anomalies were centered approximately over the regional industrial centers of Novokuznetsk (the major southern anomaly; peak NO_2_ ~ 4.25 × 10^15^ molecule/cm^2^) and Kemerovo City (the minor northern anomaly; peak NO_2_ ~ 2.90 × 10^15^ molecule/cm^2^). The maximum values of both anomalies corresponded to large urban areas with a population of ~ 0.5 million people. Moreover, the spatial gradient of NO_2_ anomaly, we identified (Fig. 3a) is consistent with that reported by Jamali et al.^16^ over this area. Specifically, the major southern NO_2_ anomaly showed the greatest NO_2_ concentration in the center, which gradually weakened toward the borders of the identified cluster of coal mines in Kuzbass (black outline in Fig. 3a). Tropospheric NO_2_ over Kuzbass was somewhat high, with an average of 3.22 ± 0.52 × 10^15^ molecule/cm^2^ within the outlined cluster of coal mines.

Most interestingly, the surge in night lights (Fig. 2) looks strikingly similar to the elevated NO_2_ over the coal basin in Kuzbass (Fig. 3a). The borders of the elevated NO_2_ did not align with the Novokuznetsk metropolitan area (white outline labeled Novokuznetsk in Fig. 3), but spatially coincided with the large cluster of coal mines. As the NO_2_ southern anomaly was unevenly distributed over the cluster of coal mines, we estimated the quantitative agreement between this anomaly and the night-light intensity, which reflects local human activity. Figure 3b reveals a moderate agreement between night lights and NO_2_ within Kuzbass coal basin (Pearson correlation coefficient, r = 0.63 at *p*-value < 0.005), thereby corroborating the association between human activity and the NO_2_ anomaly in the region. Although the correlation between the intensity of night lights over Kuzbass and the NO_2_ anomaly is imperfect (r = 0.63; Fig. 3b), this finding demonstrates that this cluster of mines is likely the main anthropogenic driver of NO_2_ emissions in the region. Notably, previous studies that considered NO_2_ from OMI and night lights^25,29^, did not find such a strong correlation.

**Figure 4:**
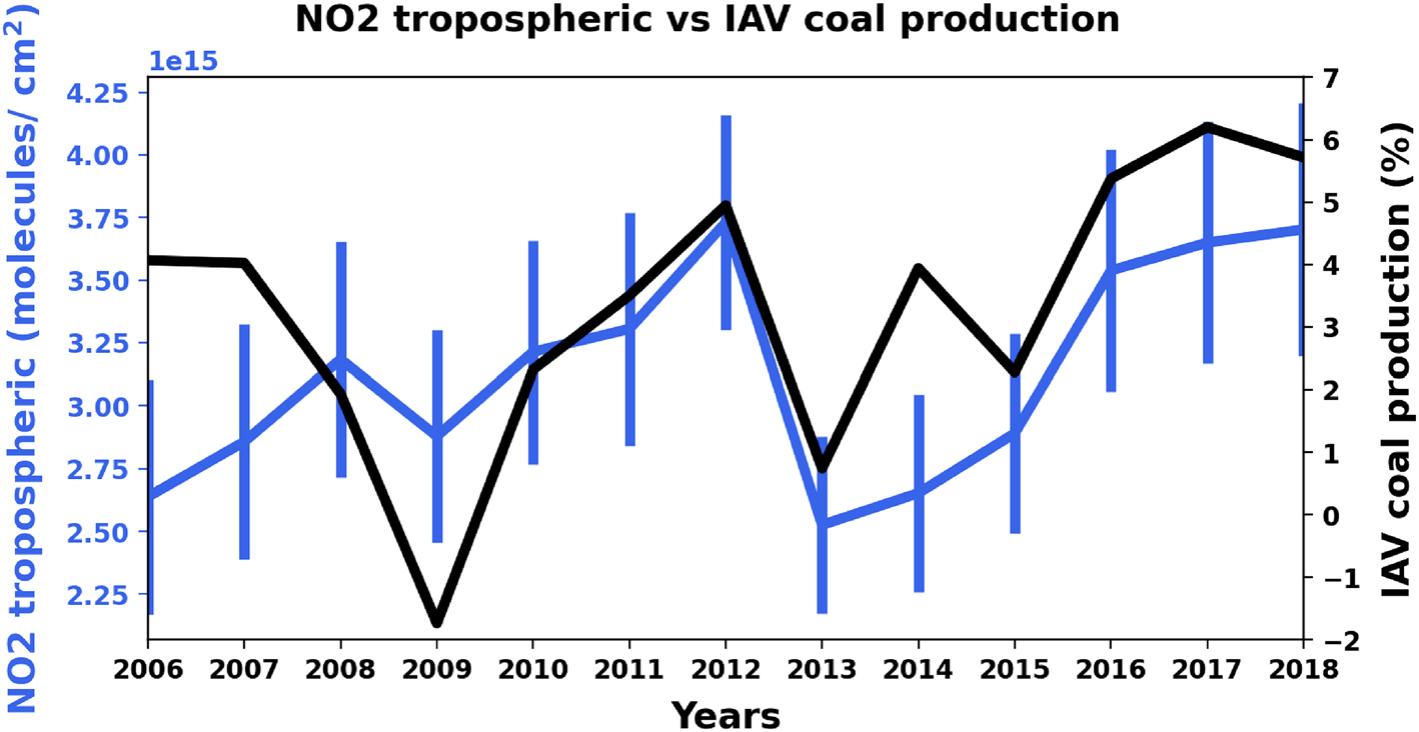
Annual average tropospheric NO_2_ (blue line with error bars representing one standard deviation) and coal production IAV (black line) in Kuzbass for the years 2006–2018 (see Table S5 in supplementary material for numerical estimates) of NO_2_ tropospheric column.

To assess the spatial association between coal mining/processing, and the NO_2_ anomaly over Kuzbass, we estimated the statistical agreement between them for 2006–2018. This period differs from that in the previous section due to the availability of coal production data. At first glance, there was no strong correlation between the annual coal production rates and annual averages of NO_2_ over Kuzbass (r < 0.50). Although NO_2_ did not exhibit any significant temporal trend in Kuzbass in 2006–2018 (*p*-value > 0.05 based on a Mann-Kendal trend test), coal production exhibited clear increasing trend in the same period (*p*-value < 0.05, slope = 5.99). As coal production is often a monotonically increasing quantity and it exhibited clear increasing trend in Kuzbass, the actual variability in production can be hidden in an inter-annual signal. Hence, we tested the inter-annual variability (IAV) of coal production, a method previously applied to similar monotonically increasing quantities such as fossil fuel emissions^30^. Fundamentally, IAV_coal_ reflects the difference (%) between an annual estimate of coal production during year *n* minus the coal production of year *n*- 1. We identified reasonable agreement (r = 0.60) between IAV_coal_ and the annual average estimates of NO_2_ over Kuzbass (Fig. 4) despite seemingly incomplete regional coverage by OMI observations during these years.

Although the statistical correlation is apparent, it was weakened by a few prominent differences in trend between NO_2_ and IAV_coal_, such as in 2007‒2008 (Fig. 4), when the world economic crisis struck most countries including Russia. IAV_coal_ fell below 0%, which is the sole decrease in coal production in Kuzbass over the 11 year study period. The decline in coal production was more rapid than the general weakening of socio-economic activity within urban areas. This phase difference was driven by the dramatic increase in the cost of transporting coal. In particular, railway transportation (99% of coal is transported from Kuzbass to other regions by train) reached 40% of the Russian coal price in 2008^31^. Moreover, declines in related industries (iron, steel, chemical, and power) abruptly constrained the supply of resources required for coal mining^31^. In contrast, activities in urban areas were not so immediately affected by these factors.

**Figure 5:**
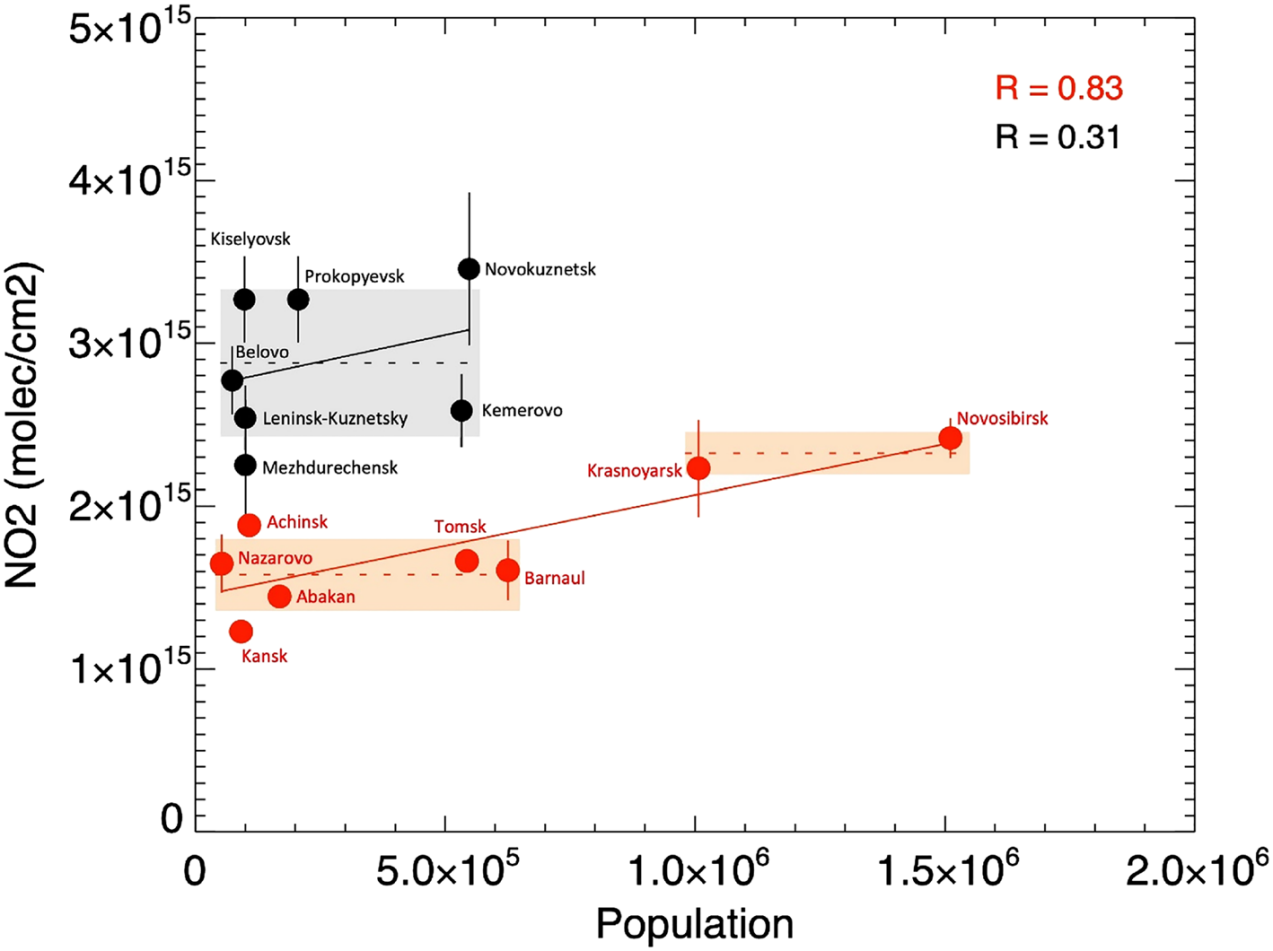
Scatter plot of mean OMI NO_2_ and population for the main cities in the Kuzbass basin (black circles) and surrounding Siberian cities (red circles) for the same time period as Fig. 4. The dot labeled ‘Kemerovo’ indicates Kemerovo city. Shaded regions highlight the standard deviation and the mean (horizontal dashed lines) of the population of the cities larger than about 1,000,000 inhabitants and lower than about 500,000 inhabitants.

The importance of coal production as one of the major drivers of NO_2_ anomaly over Kuzbass was further evaluated by analyzing statistical agreement between city-scale estimates of NO_2_ and population count from the national inventories^32^. This evaluation was based on the knowledge that in most cities, population, not coal is the main driver of NO_2_^33^. To this end, two types of cities were used in the analysis: the major cities of Kuzbass (black circles) and the neighboring major cities in Siberia (red circles) in Fig. 5. This analysis revealed two distinct patterns. First, NO_2_ over most Kuzbass cities (> 2.5 × 10^15^ molecule/cm^2^ except Mezhdurechensk) was distinctly higher, compared with other Siberian cities (< 2.5 × 10^15^ molecule/cm^2^ except Novosibirsk; the largest city of Siberia). Second, the strong linear association between NO_2_ and population was discerned for other Siberian cities (r = 0.83), thereby, confirming the common, population-related driver of NO_2_ in these cities. Notably, the correlation between OMI-based NO_2_ and population in these Siberian cities is higher than for cities in the U.S., Europe, China, and India^34^. However, despite the similar number of compared points, there was no such agreement for the Kuzbass cities (r = 0.31), indicating that population size was not the main driver of NO_2_ in Kuzbass. Figure 5, illustrating the relationship between NO_2_ and population in Siberia, indirectly suggests that the recorded NO_2_ levels in Kuzbass would correspond to a city with a population of > 2.5 million inhabitants. Overall, the combination of such high NO_2_ tropospheric concentration and the lack of relationship with population points to the existence of another driver of the NO_2_ increase in Kuzbass, unrelated to population, which might be coal-associated activities including mining, processing, transportation, and utilization of coal.

**Figure 6:**
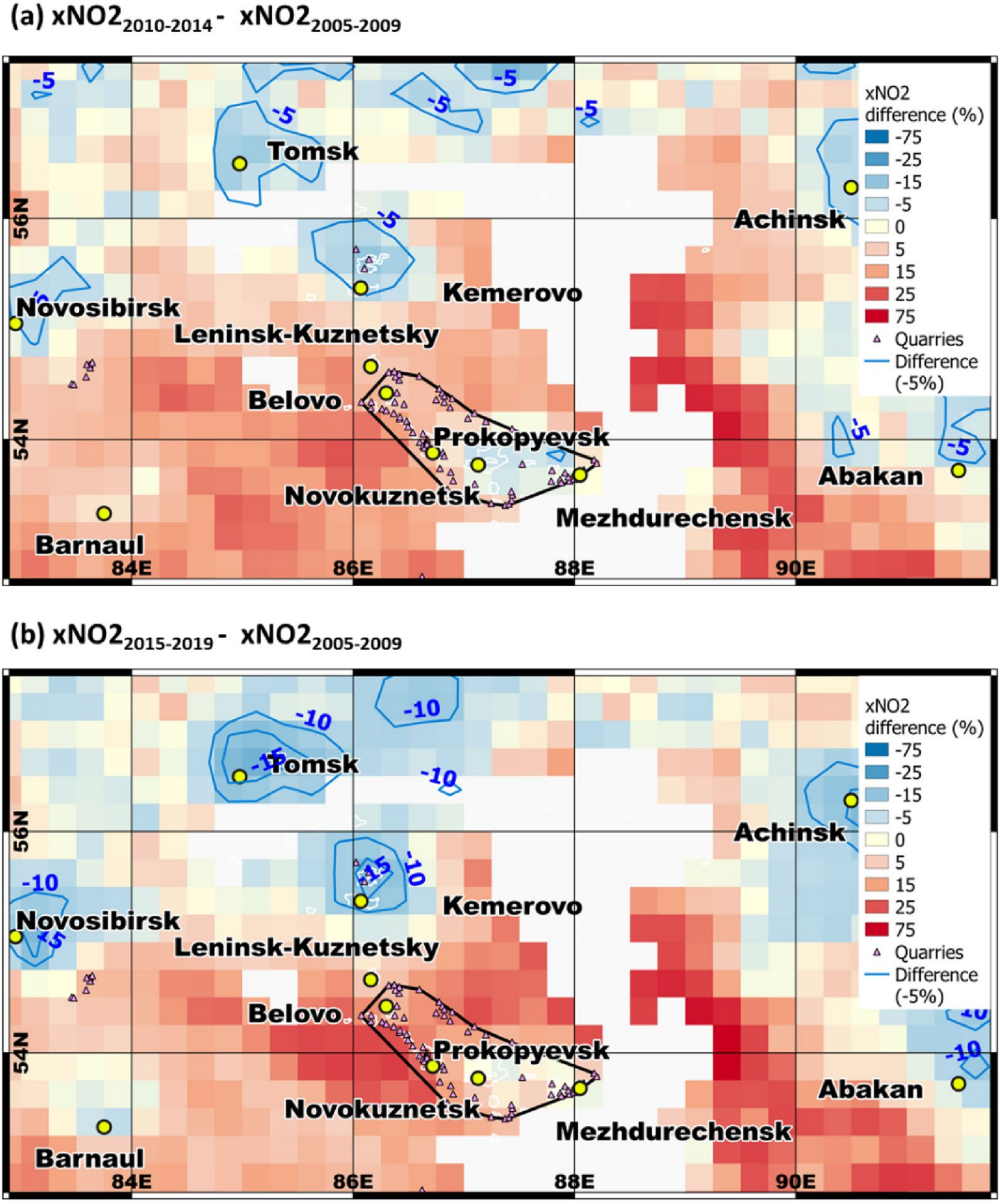
Change in average NO_2_ from 2005–2009 to (**a**) 2010–2014 and (**b**) 2015–2019. Blue shading represents a decrease in NO_2_ and red indicates an increase. Yellow circles mark the largest cities in the region, and small pink triangles indicate coal mines. The pixel size is 0.25° × 0.25°. This analysis was conducted for retrieving NO_2_ over areas with elevated NO_2_ concentrations, including cities and the cluster of coal mines in Kuzbass, whereas the hinterlands were less important for this analysis given the lower tropospheric NO_2_ over these areas (Fig. 3). To this end, the NO_2_ data from areas with relatively low NO_2_ were masked out (white areas). Areas with low NO_2_ were defined as follows: the NO_2_ concentration for a 0.25 × 0.25 grid cell is less than the mean NO_2_ concentration over Kuzbass for the period 2005–2019.

Coal mining and processing activities were likely among the main drivers of the NO_2_ anomaly over Kuzbass, given the direct evidence (the overlap between the NO_2_ anomaly and the cluster of coal mines, and the correlation between coal production and tropospheric NO_2_ over Kuzbass) and indirect evidence (tropospheric NO_2_ over Kuzbass not being related to population). This is intriguing and raises the question of whether monotonically increasing coal production affected the strength of the NO_2_ anomaly over Kuzbass. To answer this question, we relied on previous findings, which reported dramatic decreases of spaceborne-based NO_2_ (40% for some cities in the U.S.) over urban areas worldwide in 2015, compared with 2005 due to technological improvements and stricter regulations of emissions^34^. As these regulations were mostly underpinned to vehicular and stationary emissions, we tested whether the Russian NO_2_ hotspots were within this decreasing global trend of the NO_2_, by assuming that the coal mining/processing activities were not affected by these measures. If this hypothesis is correct, the NO_2_ change over Kuzbass would differ from that over the Siberian urban areas outside Kuzbass (red circles in Fig. 5). Therefore, we retrieved average NO_2_ over a broad geographic area around Kuzbass for the baseline period (2005–2009) when these regulations had not yet been enacted. We repeated this for two subsequent periods (2010–2014 and 2015‒2019) when the regulations were assumed to have affected the related emissions. Figure 6 shows the differences between each of the latter periods and the baseline period.

Interestingly, two distinctly different patterns were identified. First, the Siberian urban NO_2_ hotspots outside Kuzbass did follow the decreasing global trend as NO_2_ in 2010‒2014 has been reduced on -5% over such cities as Achinsk, Novosibirsk, Abakan, Kemerovo (are shown in Fig. 6a) and Krasnoyarsk (outside the map of Fig. 6a), compared to the baseline period. Moreover, the NO_2_ reductions have continued over these Siberian cities (reaching − 14%) in 2015‒2019, compared to the baseline period, whereas the more recent reduction of NO_2_ (− 4%) was registered over Barnaul (Fig. 6b). At the same time, the dynamics over the Kuzbass basin (and related coal quarries), except a minor regional cluster, were strikingly opposite. There was a prominent increase of NO_2_ over Kuzbass, whereas the average NO_2_ over coal quarries within Kuzbass increased on ~ 7 ± 5% and ~ 15 ± 8% in 2010–2014 and 2015–2019, respectively, compared to the baseline period. The only exception was a minor regional cluster in Kuzbass near Mezhdurechensk, where NO_2_ remained nearly unchanged in 2009–2019, compared with the baseline period (0–1% change). We suggest this was due to its topography, as the city is at the highest point in Kuzbass (532 m asl; Fig. S1), which is otherwise mostly flat (< 400 m asl). Another factor might be the lack of coal production plants within Mezhdurechensk, whereas Belovo and Kiselevsk each have two, and Prokopyevsk has one (Table S4 in the Supplementary Material). The latter factor potentially indicates that the combination of coal mining activities (i.e., mines) and coal production facilities (i.e., coal preparation plants) exacerbates the atmospheric NO_2_ anomaly in the Kuzbass cities with both coal mining and processing activities.

Novokuznetsk, which is also within Kuzbass, exhibited a slightly different pattern from the increasing NO_2_ seen over Kuzbass (Fig. 6). In particular, NO_2_ levels fell in 2010–2014 (by 4% relative to 2005–2009), and subsequently returned to the baseline level in 2015–2019. The different pattern for Novokuznetsk is reasonable, as the city is located within Kuzbass but has no coal mines (see Fig. 2) because it specializes in industrial production^28^, where ~ 62% of city enterprises produce, supply or support the production of metal^35^. Interestingly, although Novokuznetsk is not a city with coal-oriented economy, coal is actively used as input in its production of metal as conversion of most or all metal ores to usable metal is highly energy intensive. Metal production facilities use coal to provide energy and the metals are being produced by conversion of coal to coke, where both processes emit substantial NO_2_ emissions in the atmosphere. Although the data on metal production of Novokuznetsk is scanty, we analyzed Novokuznetsk inventory-based NO_2_ emissions from a previous study^36^. Notably, NO_2_ emissions from metal production of West-Siberian Metal Plant (WSMP) account for 84.2% of all the gaseous pollutants of Novokuznetsk^36^ in 2014–2018. Most importantly, we found high correlation (r = 0.76) between inventory-based NO_2_ emissions from WSMP^36^ and our annual NO_2_ tropospheric estimates from OMI and, where even higher correlation (r = 0.84) was discerned between WSMP NO2 emissions and coal production from Fig. 4. These findings clearly indicate that metal manufacturing of Novokuznetsk is based on coke-intensive input, thereby pointing on a key role of coal in these emissions.

## Discussion

The sensitivity of tropospheric NO_2_ (measured by OMI) to the mining, production, and transportation of coal in Russia’s largest coal basin (Kuzbass) was demonstrated for the first time. A major long-term tropospheric NO_2_ anomaly was revealed over Kuzbass (3.22 ± 0.52 × 10^15^ molecule/cm^2^) in the period 2005–2018, indicating substantial gaseous pollution over the region. The anomaly was attributed to the Kuzbass coal basin, based on moderate agreement identified between (1) the spatial distributions of NO_2_ and night lights originating from the cluster of coal mines and cites, as well as by (2) the correlation between inter-annual coal production and annual NO_2_ levels in Kuzbass (r ≥ 0.60). Unlike the global trend of NO_2_ reduction over urban areas (including Siberian cities), NO_2_ substantially increased over Kuzbass in the 2010–2014 and 2015–2019 periods (7% ± 5% and 15% ± 8%, respectively, relative to a baseline period of 2005–2009). The total coal production in Kuzbass was 888, 993, and 1,151 million tons in the periods 2005–2009, 2010–2014, and 2015–2019, respectively. Production in the two latter periods increased by 11% and 30%, respectively, compared with the baseline period. Remarkably, such increases seem to be strikingly proportional to our reported NO_2_ increments over the Kuzbass basin during the same period. Assuming a proportional relationship between NO_2_ and coal production, a ~ 1.0% increase in coal production is likely to cause a ~ 0.5%–0.6% increase in the NO_2_ concentration in the region, where some portion of this increase may have been offset by regulations limiting emissions.

From regional perspective, the demonstrated association between tropospheric NO_2_ with coal mining and processing activities over the largest coal basin in Russia is valuable, given the limited opportunity to otherwise assess the environmental impacts of coal-related activities in developing/emerging economies such as Russia, where official information is often inaccurate and atmospheric observations are scarce. These first estimates of substantial tropospheric NO_2_ increases over Kuzbass can encourage national and regional policy makers to formulate new pollution mitigation strategies to assess and to minimize the local population’s exposure to the adverse cardiovascular and respiratory effects of tropospheric NO_2_ and also O_3_, as NO_2_ is a precursor of O_3_. Moreover, elevated atmospheric NO_2_ may cause indirect adverse environmental effects such as nitrogen enrichment of water bodies via deposition, which compromises the safety of drinking water as well^37^.

Our findings are novel at a global level, as most existing spaceborne studies focusing on coal mining have reported increases in atmospheric CH_4_^6,7,38,39^. Although we demonstrated that spaceborne observations of NO_2_ can be utilized to attribute NO_2_ pollution to previously unreported coal mining and processing over large exploited coal basins, the hypothesis that coal mining itself can be a major source of NO_2_ emissions should be evaluated. The tropospheric NO_2_ anomaly over Kuzbass could be driven both by direct nitrogenous outgassing from coal mining (e.g., scraping and fracturing) and indirect nitrogenous emissions from coal mining and processing. Such indirect nitrogenous emissions originate from the inefficient compression-ignition diesel engines of coal mining equipment (excavators), coal transportation vehicles (haul trucks) and from nearby facilities for processing coal. Notably, the combustion of heavy fuel by the haul trucks deployed in Kuzbass has already been questioned from an ecological viewpoint^19^. Although new heavy machinery meeting Euro-3 and Euro-4 standards is being delivered to Kuzbass, there remains an abundance of inefficient diesel-fueled equipment prone to emitting NOx^19^. Moreover, as preliminarily indicated, such indirect coal emissions can stem from manufacturing of steel, which uses coke in the production, thereby generating various indirect air pollutants including NO_2_.

We encourage future studies to use Tropospheric Monitoring Instrument (TROPOMI) observations for the high-resolution analysis of NO_2_ over Kuzbass as the region is virtually unknown by the TROPOMI research community. Such studies could accurately estimate emissions from coal-related sources in Kuzbass by implementing inversions into atmospheric transport models with high-resolution spaceborne NO_2_ observations as the input. In this context, the combination of TROPOMI observations with the emission estimates from inventories can (a) disentangle direct emissions from coal mining and processing from indirect emissions originating from the inefficient fuel combustion of coal mining machinery and coke production of steel industry. Moreover, in this way, one can (b) elucidate the contributions of other human activities unrelated to coal mining to the identified NO_2_ anomaly over Kuzbass. Once these aspects are clarified, further research can estimate the coal mining-induced direct nitrogenous outgassing from Kuzbass.

## Methods

### Proxy data: anthropogenic activities and coal production in Kuzbass

As no institution has consistently reported statistics on coal production in Kuzbass, we collected fragmented data in coal production from various sources including the Administration of Kemerovo Region, the Department of Coal Mining/Production of Kemerovo Region, and the journal *Coal of Kuzbass*. We compiled coal production statistics for the available years (2006‒2018) in Table 1. For further information, see the Supplementary Information and the online compilation of references (https://gitlab.com/labzovskii/kuzbcoal/).

**Table 1:** Annual coal production statistics for Kuzbass.

Year	Coal production (mln. Tones)
2006	174.0
2007	181.0
2008	184.5
2009	181.3
2010	185.5
2011	192.0
2012	201.5
2013	203.0
2014	211.0
2015	215.8
2016	227.4
2017	241.5
2018	255.3

### OMI NO_2_

OMI is a spectrometer onboard the NASA’s EOS-Aura satellite, which is operated in the ultraviolet–visible range (visible channel is used for NO_2_ retrieval) since 15 July 2004 at sun-synchronous orbit^40^. We used the retrievals of NO2 tropospheric column, shortly referred as NO2 (Level 3 data, 30% cloud screened product called ‘OMNO2dv003’) in 2005–2018 period. The number of molecules of NO_2_ in atmospheric column were applied with only near-clear sky conditions (cloud radiance fraction < 30%). The spatial resolution of NO2 estimates is 0.25° × 0.25°, produced by the averaged observations (originally ranging from ~ 13 km × 24 km in near nadir to ~ 24 km × 160 km for the observations at the edge of a swath) over a grid cell of interest^41^. The version 3 retrieval stands out with high accuracy of retrievals of NO2 slant column density^42^. This study used the long-term averages (2005–2018) of NO_2_ as representative long-term estimates over a broad region, following previous studies^16^ and prior indications about the link between coal mining and processing activities and NO_2_ emissions^43^. The period of 2005–2018 was selected for OMI based on the availability of coal production data, namely, from the first year with full year OMI NO2 coverage until 2018, the last year for which the coal production in Kuzbass was available. The analysis was extended on one year (2005–2019) for Fig. 6 as we did not need to use coal production as a reference the analysis, shown on this figure. We also calculated annual averages of NO_2_ over the region of interest (Kemerovo Region and Kuzbass), and provided the corresponding standard deviations. The data were accessed (on 07.12.2021) using the Giovanni tool of the NASA EarthData service (https://disc.gsfc.nasa.gov/information/tools?title=Giovanni).

## Supplementary Information

Supplementary Information.

## Data Availability

The sources of the used data are mentioned in the “Methods”. Other generated data and tools are available upon request by any user; they are both freely available to any researcher wishing to utilize them for non-commercial purposes, without breaching participant confidentiality. To request the data from this study, please contact LL (labzowsky@gmail.com).
