# Supplementary material for: Spaceborne NO2 observations are sensitive to coal mining and processing in the largest coal basin of Russia
Source: Sci Rep. 2022 Jul 22;12:12597. doi: 10.1038/s41598-022-16850-8 (PMC9307612; doi:10.1038/s41598-022-16850-8)
Supplement: Supplementary file 1 — Supplementary Information. [file 41598_2022_16850_MOESM1_ESM.docx]

**SUPPLEMENTARY MATERIAL:** **SPACEBORNE NO_2_ OBSERVATIONS ARE SENSITIVE TO COAL MINING AND PRODUCTION IN THE LARGEST COAL BASIN OF RUSSIA**

**S.1 Additional information about the used data**

As mentioned in the main body of the manuscript, the information about coal production in Kuzbass (and the corresponding Kemerovo administrative region) was only fragmentarily reported over the studying period. We compiled the information from various sources including Administration of Kemerovo Region, Department of the Coal Mining/Production of Kemerovo Region, Journal “Coal of Kuzbass”, etc. All the detailed references are provided in the supplementary file 2, enclosed to this submission and in the corresponding online repository, which had been specifically created for this purpose [<https://gitlab.com/labzovskii/kuzbcoal/>]. While the accuracy and uncertainties of these estimates are unknown (a typical drawback of any inventory, not only coal production in Kuzbass), the coal production estimates can be validated by limited inventory reporting at the regional level as such as the statistics from the Ministry of Environment of Kemerovo Region [Ministry of Environment, 2020; <http://kuzbasseco.ru/005/1.5.html>]. As the regional ministry’s statistics are constrained until 2010, we have only five years to compare with our baseline coal production estimates. We found the absolute difference between the estimates does not exceed 4,0% (0.0%,0.0%,1.9%,1.8% and 4.0% for 2006, 2007, 2008, 2009 and 2010), which is fairly reasonably for the inventory intercomparison as they often disagree on much greater extent at annual scales, as mentioned in the introduction [see Guan et al., (2012) in the main list of the references].

In terms of digital elevation data, we used DEM (Digital Elevation Model) over Kuzbass from world digital elevation model ETOPO5, which had been initially introduced based on a digital dataset from land and sea floor elevations on a 5-min. latitude/longitude grid from 1988 year. We utilized ‘dem_img.zip’ file from European Environmental Agency website (<https://www.eea.europa.eu/data-and-maps/data/world-digital-elevation-model-etopo5>). The file contained the raster image that was clipped based on the Kemerovo Region administrative region shapefile (see Fig. S1 below).


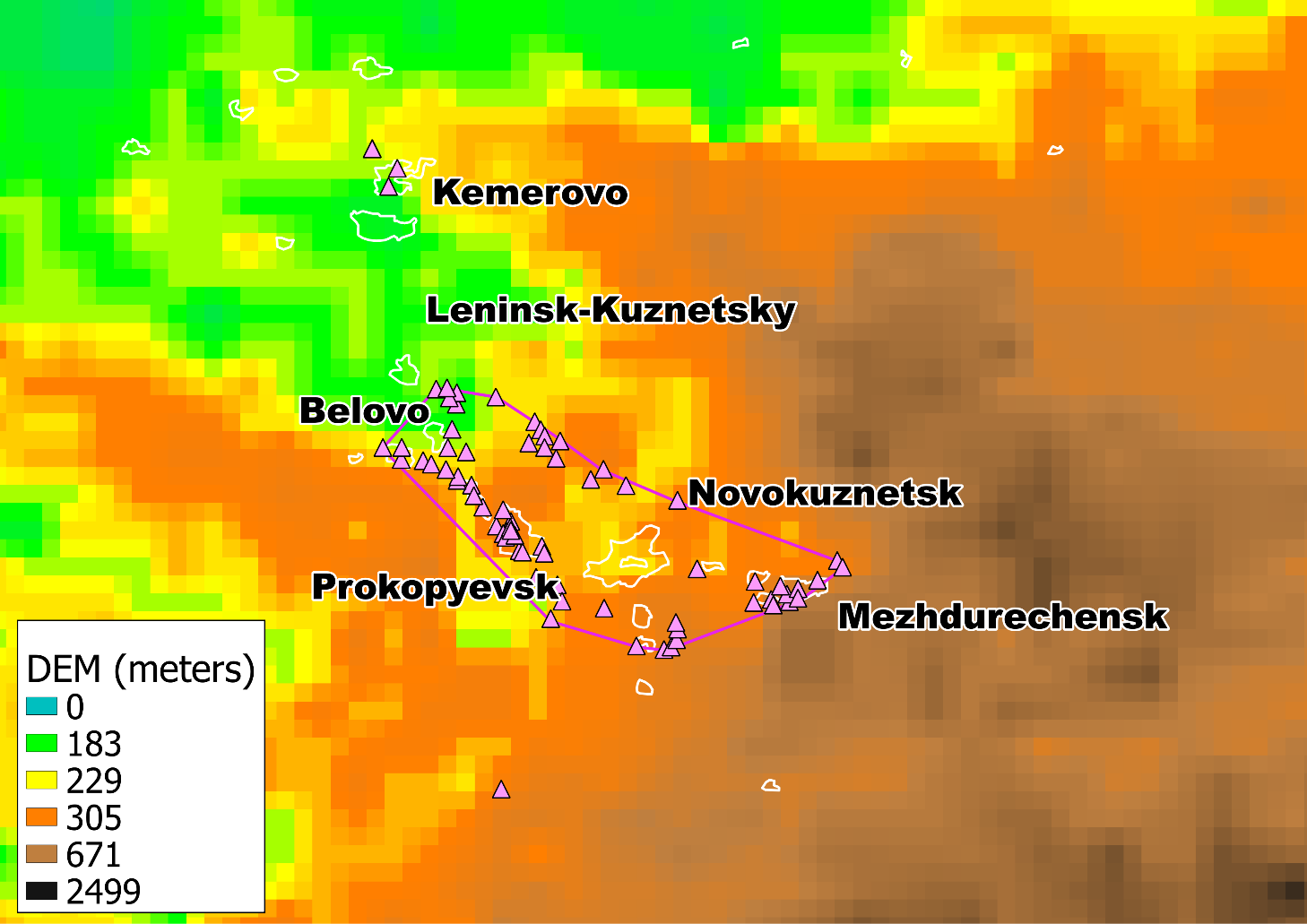


Figure S.1 DEM (Digital Elevation Model) information over Kuzbass (meters) from <https://www.eea.europa.eu/data-and-maps/data/world-digital-elevation-model-etopo5>

**S.2 Additional evidences**

Fig. S.2 below proves the visual salience of quarries with green vegetation of the Taiga ecosystem in background. Note that manual detection of coal quarries was relatively simple given abundance of Taiga-type of forest in Kemerovo region and low level of urbanization of Kemerovo Region (only two large cities of Kemerovo city and Novokuznetsk within).


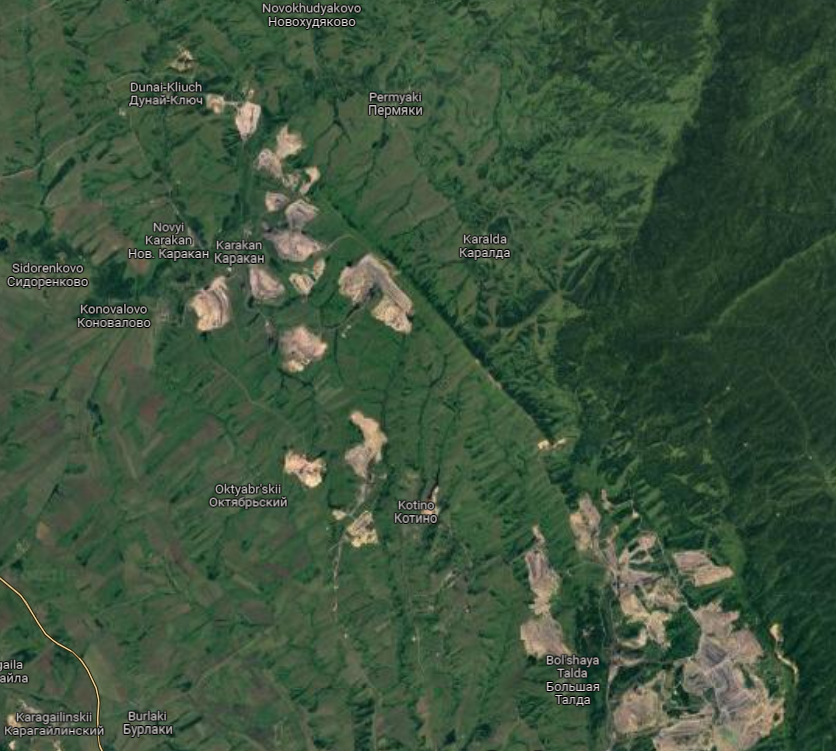


Figure S.2 The segment of the map over Kuzbass region. Sandy-grey color areas correspond to the open coal quarries. The satellite map provided by Google maps and embedded by using QucikMapServices plugin of QGIS software (0.19.29 https://nextgis.com/blog/quickmapservices/).

Fig S.3 below demonstrates contours of DEM from ETOPO5 overlapped with NO2 tropospheric column in 2005 – 2018 period. As seen, the strongest anomaly in the center of Kuzbass corresponds to ~250 m elevation (e.g., the lowest topographic area in the region).


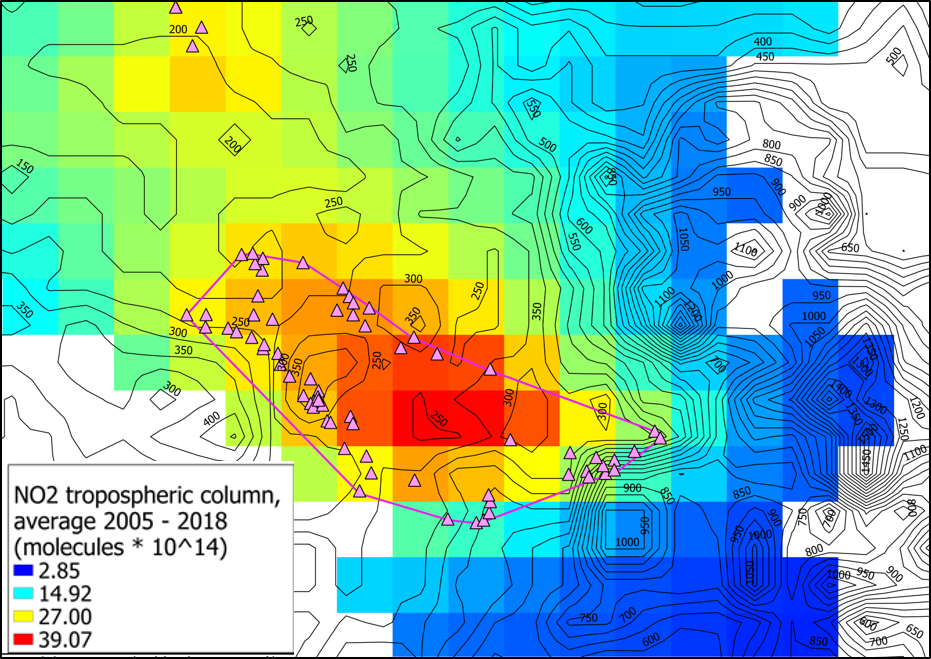


Figure S.3 Topography of Kuzbass region and NO2 tropospheric column

Table S.4 summarizes the coal mining-associated facilities in the cities of Kuzbass including

coal quarries, coal preparation plans and coal transportation hubs within these cities.

Table S.4 Coal-mining associated facilities in the cities of Kuzbass

| **City** | **Coal-Mining-Associated Facilities** |
| --- | --- |
| Kiselevsk | Coal Quarries – 4  Coal Preparation Plants – 2 |
| Belovo | Coal Quarries > 5*  Coal Preparation Plants – 2  Coal Transportation Hub – 1 |
| Prokopyevsk | Coal Quarries > 10  Coal Preparation Plant – 1 |
| Mezhdurechensk | Coal Quarries > 10 (including the largest coal quarry in Russia) |

The information about coal-related enterprises can be found in a comprehensive Russian catalogue of economic enterprises which can be classified by cities (<https://sfo.spr.ru/>)

Table S5 reports average annual tropospheric column NO2 estimates from OMI over Kuzbass in 2006‒2018, supporting the analysis, demonstrated in Fig. 4 of the main text.

Table S5 Average annual tropospheric column NO2 estimates from OMI over Kuzbass in 2006‒ 2018

| Year | Average NO2 column |
| --- | --- |
| 2006 | 2.63839E+15 |
| 2007 | 2.85749E+15 |
| 2008 | 3.18442E+15 |
| 2009 | 2.87944E+15 |
| 2010 | 3.21419E+15 |
| 2011 | 3.30528E+15 |
| 2012 | 3.73096E+15 |
| 2013 | 2.52589E+15 |
| 2014 | 2.65266E+15 |
| 2015 | 2.8908E+15 |
| 2016 | 3.53866E+15 |
| 2017 | 3.64994E+15 |
| 2018 | 3.70256E+15 |
